# The Mitochondrial Genome in Aging and Disease and the Future of Mitochondrial Therapeutics

**DOI:** 10.3390/biomedicines10020490

**Published:** 2022-02-18

**Authors:** Sanjana Saravanan, Caitlin J. Lewis, Bhavna Dixit, Matthew S. O’Connor, Alexandra Stolzing, Amutha Boominathan

**Affiliations:** 1 Division of Mitochondria Biology, SENS Research Foundation, 110 Pioneer Way, Suite J, Mountain View, CA 94041, USA; sanju.y96@gmail.com (S.S.); caitlin.lewis@sens.org (C.J.L.); bhavna.dixit@sens.org (B.D.); alexandra.stolzing@sens.org (A.S.); 2 Underdog Pharmaceuticals Inc., 110 Pioneer Way, Suite J, Mountain View, CA 94041, USA; matthew.oconnor@underdogpharma.com; 3 Wolfson School of Mechanical, Electrical and Manufacturing Engineering, Loughborough University, Loughborough LE11 3TU, UK

**Keywords:** mitochondria, mtDNA, mtDNA mutations, mitochondrial diseases, mtDNA editing, allotopic expression, gene therapy

## Abstract

Mitochondria are intracellular organelles that utilize nutrients to generate energy in the form of ATP by oxidative phosphorylation. Mitochondrial DNA (mtDNA) in humans is a 16,569 base pair double-stranded circular DNA that encodes for 13 vital proteins of the electron transport chain. Our understanding of the mitochondrial genome’s transcription, translation, and maintenance is still emerging, and human pathologies caused by mtDNA dysfunction are widely observed. Additionally, a correlation between declining mitochondrial DNA quality and copy number with organelle dysfunction in aging is well-documented in the literature. Despite tremendous advancements in nuclear gene-editing technologies and their value in translational avenues, our ability to edit mitochondrial DNA is still limited. In this review, we discuss the current therapeutic landscape in addressing the various pathologies that result from mtDNA mutations. We further evaluate existing gene therapy efforts, particularly allotopic expression and its potential to become an indispensable tool for restoring mitochondrial health in disease and aging.

## 1. Introduction

Mitochondria are found in nearly every cell type in the human body, the sole exception being red blood cells. Organelle biogenesis is semi-autonomous and involves the coordinated action of nearly 1400 nuclear-encoded proteins, including those responsible for organelle DNA (mtDNA) replication and mitochondrial fusion and fission [1,2], along with the essential 13 subunits encoded in mtDNA itself. The vast majority of these nuclear-encoded proteins are translated in the cytosol, and thus must be targeted to their mitochondrial destination, either by (1) an N-terminal mitochondrial targeting sequence (MTS), generally consisting of a positive charge and an amphipathic α-helix, which directs the protein to translocases of the inner and outer mitochondrial membranes for matrix import or (2) using poorly understood “cryptic” signals carried within their mature protein sequences, which may be recognized with the assistance of chaperones [3].

The quality and number of mitochondria in a cell are regulated by mitochondrial biogenesis (fusion and fission) and turnover (largely governed by mitophagy, a mitochondria-specific form of autophagy) [4]. Fusion of mitochondria, wherein undamaged mitochondrial components are combined, not only defends the cell against internal build-up of oxidative damage, but is also used to assemble an intracellular network for energy distribution throughout the cell [5]. Conversely, mitochondrial fission is necessary for isolating damaged mitochondria from the network to be targeted for mitophagy and the process can, in principle, also be controlled to select against mutated mtDNA in heteroplasmic cells [6,7]. Factors that impede efficient removal of damaged mitochondria increase mitochondrial ROS production and mutant mtDNA load, which ultimately decreases cell survival [8].

## 2. mtDNA and Its Role in Mitochondrial Function

Despite organelle-level protective mechanisms, mtDNA remains especially vulnerable to damage and accumulation of mutations. Quality and quantity of mtDNA is closely linked to mitochondrial function [9,10] and its location adjacent to the OXPHOS machinery exposes the genetic material to higher risk of mutagenic events [11]. In humans, the remnant mitochondrial genome contains 37 genes, encoding for 13 proteins, 22 tRNAs and 2 rRNAs [12]. All 13 proteins synthesized from mtDNA are integral subunits of the 5 enzyme complexes which comprise the oxidative phosphorylation (OXPHOS) relay. mtDNA is more susceptible to mutation than its nuclear counterpart due to several factors, including its high replication rate and errors therein, the paucity of effective DNA repair mechanisms within the organelle, an absence of the canonical protective proteins observed in nuclear DNA, such as histones, and its proximity to DNA-damaging ROS byproducts of the oxidative phosphorylation relay [13,14,15].

The genes and protein products controlling mtDNA replication are all synthesized from nuclear DNA and can be grouped according to three main functions: (1) the DNA replication process itself, (2) maintenance of the nucleotide balance within the organelle, and (3) mitochondrial homeostasis mechanisms, such as fusion and fission. In theory, a mutation or impaired function in any of these component genes can thus result in compromised mtDNA synthesis.

There are between 5 and 10 copies of mtDNA in each mitochondrion, and depending on the energy burden, a cell may contain hundreds to thousands of mitochondria, adding up to a very large mtDNA copy number [16]. Acquisition of aberrant mtDNA from the mother and/or selective amplification of mutated mtDNA during embryogenesis and later during the lifespan of the individual are causative for several known mitochondrial diseases. As mtDNA propagates independently of the cell cycle, this can result in replication of mutated mtDNA alongside wild-type copies, which then segregate randomly and asymmetrically with the mitochondrial network during cell division. High mutation frequency combined with the large number of mtDNA copies in each cell leads in most cases to both wild-type and mutated mtDNA coexisting in a heteroplasmic condition [17,18]. There is however evidence for clonal expansion of some mutant mtDNA in postmitotic cells which then become homoplasmic for a range of large deletions with age [19]. Aberrant ratios of mtDNA heteroplasmy have been implicated in numerous pathologies, including both inherited mitochondrial encephalomyopathies, and acquired conditions, such as type 2 diabetes mellitus, aging, cancer, and neurodegenerative diseases [17,18]. There is often a direct correlation between the level of mutant mtDNA heteroplasmy and the severity of the phenotype, though for many diseases caused by mtDNA mutations, the heteroplasmy must cross a threshold to cause clinically recognized symptoms [17,20].

Specific mutations in the 13 oxidative phosphorylation genes are known to cause a host of mitochondrial diseases and disorders, and their study offers mechanistic insight linking mutation with functional impairment. It is observed that mtDNA mutations which cause structural changes in OXPHOS subunits disrupt the electron transfer relay, resulting in inefficient energy production. Inefficient transfer can, in turn, generate superoxide byproducts, resulting in increased ROS and reactive nitrogen species (RNS), causing a chronic state of cellular stress. This cascade can overwhelm mitochondrial protective stress responses, such as fission and fusion, proteostasis, and mitophagy, thereby allowing continued exposure of mtDNA to mutagenic agents while permitting damaged mtDNA and defective proteins to persist—all of which contribute to further damage accumulation and functional decline [21].

The consequences of mtDNA mutations are often most apparent in cells and tissues that have a high energy demand and rely heavily on OXPHOS for metabolism, like the central and peripheral nervous systems and muscle tissue. mtDNA mutations affecting these tissues constitute a heterogenous group of diseases, broadly categorized as mitochondrial encephalomyopathies, of which MERRF syndrome (myoclonic epilepsy associated with ragged-red fibers) and MELAS syndrome (mitochondrial encephalomyopathy, lactic acidosis, and stroke-like episodes) are two well-studied examples. Both of these syndromes are caused by mutations in tRNAs and OXPHOS proteins produced from the mitochondrial genome [22,23,24]. mtDNA mutations in tRNA_Leu_, ND1, and ND4 are known to cause MELAS [25,26,27,28], while the A8344G mutation in tRNA_Lys_ has been implicated in MERRF [29]. Other well-studied mtDNA diseases affecting ATP synthesis include LHON (Leber’s hereditary optic neuropathy, due to mutations in ND1, ND4, ND4L, or ND6 genes) [30,31,32,33,34], Leigh’s syndrome (with mutations in ND3, ATP6, and ATP8) [35,36,37], and NARP (neuropathy, ataxia, and retinitis pigmentosa, with mutations in the ATP6 gene) [22,38,39,40]. These diseases can be difficult to treat because patients often present with disparate symptoms and severity, even when harboring the same mutations [41,42].

Owing to the unique susceptibility of mtDNA to mutation [43,44], spontaneous alterations also arise within somatic cells and further accumulate with age [45,46,47,48]. As stated, mitochondrial DNA is prone to acquire deletions with age; in particular, it is observed that a rising number of cells acquire large deletions overlapping the gene for nicotinamide adenine dinucleotide dehydrogenase (NADH) subunit 4 (ND4) [49] which expand clonally and become homoplasmic in the affected cell [19]. The absence of any wild-type mitochondrial genomes in such cells abrogates remediation through mitophagy and, in fact, the mechanism of clonal expansion of such large deletions may itself involve defective mitophagy [50,51]. Several large deletions and point mutations also have documented associations with age-related disorders, such as Parkinson’s disease [52], Alzheimer’s disease [53], and sarcopenia [19,54]. Acquired mtDNA mutations have also been implicated as drivers of late-onset neurodegenerative conditions, including Huntington’s disease, amyotrophic lateral sclerosis (ALS), hereditary spastic paraplegias (HSP), and spinocerebellar ataxias (SCA) [55]. While specific mtDNA mutations have yet to be associated with many of these diseases, cytochrome b dysfunction has been implicated in Parkinson’s and cytochrome c oxidase deficiency is associated with SCA [56,57].

### Importance of mtDNA Maintenance in Aging

Although it is clear that dysfunction in mitochondrial metabolism is the cause of several inherited and acquired diseases, aberrant mitochondrial function has also been linked to the physiology of aging itself. Age-related changes in mitochondrial metabolism may take several forms, including a decreased number of mitochondria, declining rates of ATP synthesis, or reduced oxidative capacity [58,59]. These changes, in turn, influence critical mitochondrial functions, such as maintenance of the chemical and electrical transmembrane potentials of the inner membrane, electron transport chain subunit functions, and the transport of critical substrates and metabolites into and out of the mitochondria [60]. mtDNA mutations disrupting these processes, particularly those which decrease reliance on OXPHOS function, are also thought to promote tumor survival in many cancers by facilitating evasion of apoptosis and hypoxia-driven cell death [61,62]. Interestingly, oncocytomas comprise a distinct tumor subclass affecting endocrine tissue, characterized by non-silent loss-of-function mtDNA mutations and the absence of early driver mutations in the nucleus. Sequencing of oncocytic tumors has revealed disruptive mutations in all seven mtDNA-coded subunits of Complex I, as well as in Complex IV subunits and in ATP6 [63,64]. While many such oncocytic tumors are benign, the loss of complex I function has been implicated as an early driver in thyroid oncocytomas (Hürthle cell carcinoma), which are highly aggressive [65]. Indeed, somatic mutations in Complex I and Complex IV subunits are among the most frequent aberrations identified in the cancer-associated mtDNA landscape, which is comprehensively surveyed in several recent reviews [64,66].

In addition to changes directly resulting from mutations, decreased expression of mtDNA-encoded genes may also contribute to age-related decline in mitochondrial function [67]. Reduced expression may be due to decreased transcription levels or mRNA instability, either of which would be exacerbated by decreased mtDNA copy number [59,67]. The lower number of mRNA templates directly affects protein expression and OXPHOS complex assembly. The impact of aging on mtDNA quantity and gene expression may also be traced back to oxidative damage that accumulates within mtDNA and resultant base deletions and point mutations [67]. A study investigating mtDNA heteroplasmy and copy number in 1511 women between 17 and 85 years old showed that mtDNA heteroplasmy increases with age and mtDNA copy number decreased by an average of 0.4 copies per year in mitochondria of isolated peripheral blood mononuclear cells (PBMCs) [68]. Thus, augmenting mtDNA quality and quantity during the lifespan may help counteract the downstream effects of dysfunctional mitochondria and slow down the aging process [68]. Below, we discuss the various strategies to address pathologies arising from mtDNA mutations with special reference to gene therapy and current mtDNA editing technologies.

## 3. Mitochondrial Therapeutics

The complicated nature of mitochondrial biology makes it challenging for clinicians to treat patients diagnosed with mitochondrial diseases. Few effective treatment strategies exist to manage symptomatic patients, and none are considered curative. For adults and children with non-lethal disease manifestations, therapeutics have historically been limited to symptom management using nutraceutical supplements, and lifestyle interventions, such as dietary restriction and exercise; more severe mutations generally result in embryonic or early-life lethality.

High-throughput technologies have led to the identification of small-molecule candidates that can alter the redox balance, some of which hold promise for ameliorating patient symptoms. For example, Idebenone, a CoQ10 derivative, is the first line of treatment in LHON patients [69]. EPI-743 (α-tocotrienol quinone) and RP103 (cysteamine bitartrate) are two other small molecules in clinical trials [70] for their potential therapeutic application in several other mtDNA genetic disorders. The status of these and other pharmacological interventions have been recently reviewed [71]. In addition to small-molecule therapeutics, several groups have studied the effects of manipulating TFAM (a key activator of mitochondrial transcription that also participates in mitochondrial genome replication) [72] by inhibiting the mTORC1/S6 kinase signaling pathway to increase mitophagy [73]. Manipulation of SOD2 (mitochondrial superoxide dismutase) [74,75] is also being explored as a means to mediate the symptoms of LHON caused by the m.G11778A mutation. Though additional small molecule candidates continue to be identified, each of these treatments offers limited therapeutic potential and aims only to lessen the severity of patient symptoms or slow disease progression.

In recent years, several additional approaches have evolved, aiming for curative therapeutic potential and even prevention of germline transmission. Perhaps the most promising potential disease-modifying therapy for a congenital mitochondrial disorder currently in clinical use is allogeneic hematopoietic stem cell transplantation (AHSCT) for children with mitochondrial neurogastrointestinal encephalomyopathy (MNGIE), an extremely rare mitochondriopathy caused by mutations in the TYMP gene. This gene encodes the enzyme thymidine phosphorylase, and loss-of-function mutations result in systemic accumulation of thymidine and a range of disabling symptoms. In case studies and small, uncontrolled ongoing pilot trials, AHSCT reduced systemic exposure to thymidine and results showed significant improvements in quality of life and functional status in a subset of MNGIE patients (clinicaltrials.gov, NCT 02427178 accessed on 9 August 2021). The treatment is limited in availability by the need for immunologically matched donors, however, and entails ongoing immunosuppression, and even with treatment 63% of patients still perish from the condition [76,77]. The pathologies associated with most congenital and disease-associated age-related mitochondrial mutations are, however, not similarly linked to excessive production of a specific metabolite and occur in a cell-autonomous way in less-dispensable cell types, such as heart muscles and neurons, so this promising example is not a suitable strategy for generalized mitochondrial disease(s).

The emerging role of mitochondrial health in fertility has led to more active research into both preventative and curative treatment options, including mitochondrial replacement technologies [78] and gene editing approaches [79,80], such as with directed nucleases [81] or nucleic acid therapies [82,83]. Strategies to directly manipulate mtDNA sequences and thereby shift heteroplasmy levels have also been attempted using sequence-specific DNA editing enzymes, such as zinc-finger nucleases and TALENs. Termed “mitoREs” (for mitochondria-targeted restriction endonucleases), the first of these strategies involves importing specific DNA restriction enzymes to the mitochondrial matrix to cut unique sites introduced by mtDNA mutations [84,85,86]. However, this approach has limitations in that the acquired mutation must harbor a specific restriction site to distinguish mutant and wild-type mtDNA copies, and efficiently introducing restriction enzymes into target cells and into mitochondria still relies on challenging mitochondrial protein delivery techniques or equally complicated advanced gene therapy approaches. Advancements in the identification of sequence-specific DNA recognition proteins have led to the development of next-generation mitoREs along with strategies exploiting custom Zn-finger nucleases and TALENs to cut specific mtDNA mutations [84,85,86]. More recently, certain base-editing enzymes, such as cytidine deaminases, have also been used to modify specific sequences in the mitochondria [87]. Modified base-editing enzymes and methods for shifting heteroplasmy remain active areas of research and have been extensively reviewed [88,89,90].

All of the above DNA editing strategies are amenable only to a small subset of mutations in mtDNA. The use of CRISPR to target mtDNA is being explored [91,92], although this application of the technology remains hindered by our limited knowledge of RNA import into mitochondria and how one might achieve sufficient guide RNA (gRNA) import to confer specificity of cleavage. The importation and dimerization of a large protein, such as CAS9, to the mitochondrial matrix presents an additional challenge. One proposed strategy has been to employ different Cas nucleases, such as the smaller Type V Cas12a nuclease of *Lachnospiraceae bacterium* ND2006 (lb), in conjunction with a mitochondria-targeting gRNA aptamer [93]. gRNAs designed for mitochondrial matrix import have also been recently reported, accomplished utilizing PNPase (polynucleotide phosphorylase), a 3′→5′ exoribonuclease and poly-A polymerase that regulates the import of nuclear-encoded RNAs into the mitochondrial matrix [94,95]. These authors were able to show a ~75% reduction in mtDNA in mouse embryonic fibroblasts using an RP-gRNA targeted for the 11205G sequence in the mt-ND4 gene. Thus, although our ability to manipulate and utilize gene-editing tools is rapidly growing, in addition to technical hurdles associated with the therapy itself, numerous challenges to the above DNA editing strategies persist, making it unlikely that such an approach can offer broad curative potential.

## 4. Allotopic Expression

### Allotopic Expression of mtDNA Genes to Correct Underlying mtDNA Damage

**Figure 1:**
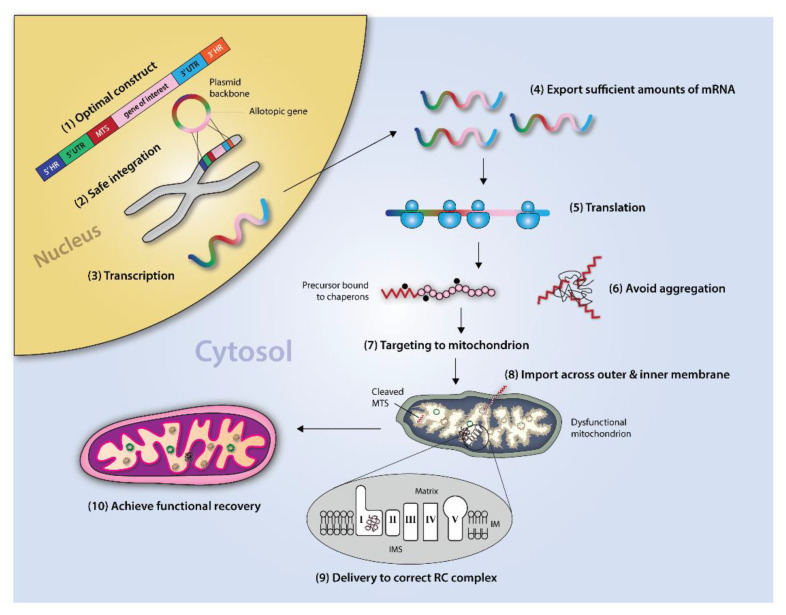
Schematic for Allotopic Expression. The various steps involved in the successful implementation of the allotopic expression strategy are depicted, beginning with design of the optimal DNA expression construct and ending with incorporation of the exogenous protein into the correct RC complex. HR: homologous region, UTR: untranslated region, MTS: mitochondrial targeting sequence, IMS: intermembrane space, IM: inner membrane, RC: respiratory chain.

The concept of allotopic expression (AE) originated from work by Nagley and colleagues in 1985 [96], wherein yeast ATP8 was recoded to the nuclear DNA code and expressed cytosolically for use as a research tool to elucidate mitochondrial function. Dozens of AE studies ensued in the following decades in an effort to gain mechanistic understanding of the organelle (summarized in Table 1). While multiple studies have found that eukaryotic ATP8 and ATP9 of *N. crassa* can be effectively recoded and expressed cytosolically [97,98,99], the basic mtDNA recoding approach has otherwise been largely unsuccessful for nuclear expression of other OXPHOS subunits, leading many to question the general feasibility of the AE strategy as a potential therapeutic approach [99,100]. It is generally accepted that design features, such as nuclear recoding and addition of a targeting signal (MTS), are minimal requirements for AE; however, researchers also agree on several likely mechanisms limiting successful allotopic expression for other mtDNA genes (Figure 1).

## 5. Circumventing Biological Roadblocks

### 5.1. Mitochondrial Targeting

One feature common to all allotopic constructs currently reported is the addition of a mitochondrial targeting sequence (MTS) from a nuclear-encoded mitochondrial gene to direct the expression product to the mitochondrion. MTSs are similar to ER-targeting signals and are predominantly found at a protein’s N-terminus, allowing recognition of the nascent chain by the organelle’s import machinery [101]. While N-terminal MTSs generally share characteristics, including presence of hydrophobic residues and an amphipathic alpha-helix structure, they are variable in length and cleavage properties. Several groups have probed the efficacy of different N-terminal extensions for import of mitochondria-destined cargo [98,99,102,103,104,105], and while most MTSs have shown the ability to transport passenger proteins to the mitochondria, efficiency of matrix translocation varies between MTSs tested and is influenced by the protein cargo. Additionally, many imported proteins fail to achieve post-import cleavage of the MTS, the requirements for which are yet to be fully understood. Incomplete translocation, an altered membrane orientation, or other disruptions to normal ETC complex assembly may further hinder successful allotopic expression.

### 5.2. Probing the Hydrophobicity Threshold

One proposed reason for inefficient import of allotopically expressed proteins is the high hydrophobicity of these 13 OXPHOS subunits, all of which exist in complexes embedded in the mitochondrial inner membrane (MIM) [99]. In fact, one widely believed hypothesis is that the hydrophobic nature of these proteins could have forced the retention of their encoding genes in mtDNA, where translation within the mitochondrial matrix places the nascent chains in direct proximity to the destination membrane. Translation from within the matrix allows subunits to be inserted into the inner membrane co-translationally, thereby avoiding aberrant hydrophobic folding or aggregation in the cytosol that may cause import incompetence. Hydrophobicity has proven to be a challenge post-translationally for several subunits. For example, cytochrome b (CYB), the sole mtDNA-encoded subunit of Complex III, remains encoded in the mtDNA of all known eukaryotes. AE attempts have repeatedly failed, with evidence that exogenous recoded CYB forms cytosolic aggregates, precluding mitochondrial import [99].

### 5.3. Coupling for Co-Translational Import

One strategy employed in the hope of circumventing temporospatial import limitations has been the inclusion of various elements of untranslated regions (UTRs) from nuclear genes encoding other proteins of the respiratory chain. In yeast, it is well-established that the mRNAs of many proteins destined for the mitochondria localize to ribosomes at the outer mitochondrial surface, suggesting that a co-translational import mechanism may exist for such nuclear-encoded proteins [136,137,138]. While the Clueless (CLUH) and Pumilio-family (Pufp) RNA-binding proteins have an identified role in mRNA localization in lower eukaryotes [139,140], an analogous mechanism has yet to be identified in mammalian cells, and no consensus mRNA “zip code” sequence has been identified. Nonetheless, it is postulated that cryptic localization signals may exist in the 5′ and 3′ UTRs of some mitochondrially destined protein genes. Several research groups have utilized this framework in an attempt to improve mitochondrial targeting of exogenously expressed proteins by means of transfactor recruitment, including unpublished experiments from our own lab, but thus far the role of UTR regions remains ambiguous. The 3′ UTRs of ATP2 [97], COX10 [121,122], or SOD2 [113], all of which are nuclear genes with mRNAs that localize to the mitochondrial surface, have been examined and employed in this context.

### 5.4. Piecewise Import

In order to circumvent cytosolic aggregation, Claros et al. attempted the piecewise expression of individual or pairs of helices of the mature CYB protein, a strategy which has been successful for hydrophobic prokaryotic membrane proteins, such as bacteriorhodopsin [120]. Studies of COX2 in yeast determined that this Complex IV subunit could be effectively expressed and imported upon replacement of hydrophobic residues in transmembrane segments of the protein [97,133,134]; however, despite achieving import, expression of the modified protein failed to restore function of the complex, suggesting that merely achieving import to the matrix does not ensure functional incorporation of the expressed protein. Studies in the mammalian ATP6 and COX3 genes also showed similar results. While protein products with apparent reduced hydrophobicity were imported into mitochondria, they were unable to achieve functional rescue [115].

## 6. Genetic and Molecular Characteristics of mtDNA-Encoded Proteins Present Inherent Challenges for Successful AE

Despite these and other efforts, constructs for many other subunits have hitherto failed to express at all, especially upon stable selection—that is, integration of the mitochondrial gene copy into the nuclear genome, rather than expression from a plasmid. As the mitochondrion is a remnant of an early endosymbiont, its highly conserved, retained genome bears similarity to those of prokaryotes and uses a coding sequence and codon usage frequencies that diverge from those of nuclear genes [124].

**Figure 2:**
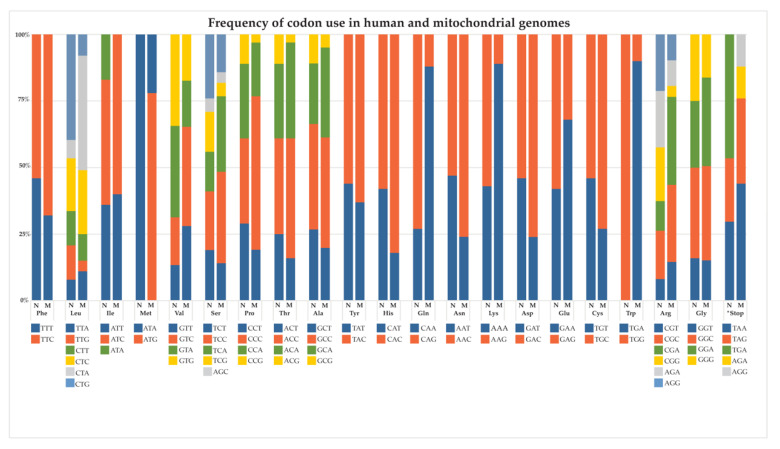
Frequency of codon use in human nuclear and mitochondrial genomes. Left column: nuclear codon usage; right column: mitochondrial codon usage for respective amino acids.

With few exceptions, AE studies have employed “minimally recoded” mitochondrial genes, wherein the only codons changed in the mtDNA sequence are those necessary to maintain the amino acid sequence using cytosolic translation machinery. As with the non-coding regions in the nuclear genome, however, we continue to uncover the influence of synonymous codon substitutions on protein abundance, structure, and function; therefore, it is likely that mitochondrial genes require coding sequences optimized for nuclear expression in addition to being recoded to express the same amino acids. Figure 2 depicts the frequency of codon use in nuclear and mitochondrial genomes. Prophetically, the very first studies of AE by Nagley and colleagues in 1985 [96] employed this very principle, through manual assembly of the yeast ATP8 sequence using codons considered ideal by nuclear expression standards, a consideration forgone by nearly every subsequent AE study over the next several decades. 

Recent work in our lab revisited this theory [116,124], and while codon optimization does not address the challenges associated with mitochondrial targeting, import, or hydrophobicity of the encoded proteins, results indicate that optimizing the gene sequence for the expression system greatly enhances our ability to translate mitochondrial genes using the nuclear machinery. In every case, the codon-optimized constructs expressed discernible protein products that associated with mitochondria in vitro, unlike their recoded counterparts. Furthermore, several of the Complex I genes (ND1, ND2, ND3, ND4, ND4L, and ND6), as well as COX2 from Complex IV and ATP8 from Complex V, were successfully expressed stably in mammalian cells using this approach [124]. Co-expression of Complex V mtDNA genes ATP8 (codon-optimized) and ATP6 (recoded) unequivocally rescued a severe phenotype in a patient cybrid cell line null for the ATP8 protein [116].

There are potential limitations and pitfalls to consider when optimizing codon usage in recombinant proteins meant for therapeutics, such as depleting specific tRNAs, the introduction of cryptic translation start sites [141], or the corruption of information encoded in the original mRNA coding sequence [142,143,144]. Another important factor is the impact of introducing such foreign genes into the nuclear environment and/or proteins into the cytosol, as altering gene sequences for optimal translation may also generate nucleotide and peptide sequences that can elicit host immune responses.

Gene dosage and copy number are other important considerations, as expression of mtDNA subunits is tightly coordinated with nuclear gene expression in a tissue- and condition-specific manner, with carefully regulated nuclear-to-mitochondrial subunit stoichiometry. As mitochondrial content and mtDNA copy number vary amongst cells and tissue types, nuclear expression of mtDNA genes must result in a transcriptional response appropriate for the mitochondrial load, which also fluctuates depending on energy requirements, substrate availability, and cellular signaling. Placing allotopic genes under the master regulators of mitochondrial gene expression, such as NRF1 and NRF2 or their upstream regulator PGC1alpha [145,146], might be a way to modulate and coordinate their expression with the rest of the OXPHOS subunits. Alternatively, placing the AE gene under the promoter of another gene in the same complex could similarly coordinate expression of the AE gene with other genes required for the function of its complex.

### Paucity of Animal Models to Validate Allotopic Expression 

Additional technical limitations must be considered in the implementation of mitochondrial gene therapy. Studies probing the utility of AE for mitochondrial rescue are challenged by the lack of good animal models. Genetic tools widely used to elucidate nuclear gene function, such as targeted knockouts, siRNA-mediated gene silencing, and site-directed mutagenesis, are non-viable options for mtDNA genes, which are sequestered within the organelle and therefore inaccessible. Thus, the study of mtDNA-mediated dysfunction is largely reliant on decades-old methods, mainly the use of patient-derived cybrid cell lines with specific mutations. The use of patient-specific cybrid cell lines, however, carries its own disadvantages, as most cybrid lines contain shifting ratios of wild-type and mutant mtDNA, i.e., heteroplasmy, which result in variable cellular and organismal phenotypes and weaken claims of rescue through AE.

Numerous challenges have also frustrated efforts to generate meaningful animal models of mitochondrial disease, especially for systems with loss-of-function mutations. Due to the critical function of OXPHOS subunits in respiration and metabolism, mitochondria with mutations that severely disrupt ETC function are often not viable, consistent with selection against such mutations in the maternal line preventing generation of true organismal research models of primary mitochondrial disease. This phenomenon has been demonstrated in animal models; for example, transmitochondrial mice with the dual mutation T6589C (COX1) and 13885insC frameshift mutation (ND6) genes, respectively, at equivalent levels lost the more severe ND6 13885insC frameshift mutation completely within four generations [147]. Efforts using enucleated human cybrids and mouse pronuclear cells to generate mouse models for the 4.7 kb “common deletion” that overtakes a rising number of postmitotic cells with age failed to achieve progeny beyond F3 [148]. Available systems therefore include the study of naturally occurring mild mutations with less severe phenotypes, such as those observed due to missense mutations in the ATP8 [149] and ND6 genes [150]. Researchers have also employed co-expression of mutant allotopic genes over WT versions, as in the case of ND4 [151] and ATP6 transgenic mice [117,151], in attempts to recapitulate specific disease states, such as LHON and NARP. Mouse models have further been used to study comparable phenotypes due to mutations in nuclear-encoded proteins involved in mitochondrial biogenesis, such as TFAM [152], PolG [153], Twinkle [154,155,156], and SURF-1 [157]. Such “mutator” mice are attractive models of accelerated mutation load that may mimic the accumulation of mtDNA mutations with age but are poor systems for testing and optimizing single-gene therapies. A comprehensive list of mouse models used to study OXPHOS deficiency due to nuclear-encoded OXPHOS subunits and other mitochondrial genes can be found in the following reviews [158,159]. Recent advances in generating induced pluripotent stem cells (iPSCs) from primary cells of affected patients has also led to the creation of model cell lines with specific mtDNA mutations [160,161,162]. However, PSCs can exhibit altered mitochondrial and metabolic profiles because of the switch from glycolysis to oxidative phosphorylation upon differentiation. Furthermore, individual PSCs display considerable heterogeneity in mutation loads that can impact their utilization in disease modeling and as drug screening platforms [163,164].

## 7. Allotopic Expression Has Been Demonstrated In Vivo

Despite these difficulties, some animal studies targeting the ATP6 and ND4 genes using AE have shown promising data [128,165,166,167]. This suggests that if the more direct limitations to AE of individual subunits are overcome, gene replacement therapy could be a viable option to improve mitochondrial function. As of now, to our knowledge, only the ND4, ND6, and ATP6 genes have been demonstrated in animal models for allotopic expression. ND4 has been exclusively studied in the context of LHON, and AE constructs have been examined for their ability to alleviate optic nerve damage and rescue visual acuity by restoring OXPHOS function and ATP levels. Cwerman-Thibault et al. [128] modified the ND4 gene with elements of the human COX10 mRNA to improve the delivery/import of the protein to mitochondria and showed that AAV2-mediated delivery of ND4 is expressed for up to 12 months in rats engineered to carry the G11778A LHON mutation. In addition to adjusting the translation code for nuclear expression, codon usage for the non-universal codons was optimized. Guy et al. have shown that allotopic expression of the human ND4 gene in mice, administered via intravitreal injection, rescues visual dysfunction and prevents the LHON phenotype caused by the G11778A mutation [125,126]. This group used the recoded ND4 for nuclear translation and the cytochrome oxidase subunit 8 (COX8) MTS to target the protein to mitochondria. Similarly, mutant versions of the ND4 and ND6 genes have been expressed using mitochondria-targeted AAV, to induce LHON phenotypes in mouse models [151,165,166,168]. Dunn and Pinkert generated two versions for the nuclear-recoded mouse ATP6 gene: a wildtype and mt8993T > G that causes Leigh’s syndrome and NARP in humans [117,167]. They injected plasmids harboring mitochondrial transgenes under the EF1-alpha promoter and the COX VIII MTS directly into mouse embryos. Their study outlines the design of the allotopic expression system in this mouse model, from construct design to animal genotyping. However, the various experiments used to characterize the phenotype of the rescue mice gave mixed results and failed to definitively show an improvement in mice carrying the ATP6 mutation [117] or to demonstrate robust expression of ATP6 in any of the tissues examined. Perhaps if those experiments were repeated using modern methods, including codon optimization, the in vivo expression of ATP6 would be more robust.

### A Safe Harbor Expression System for Allotopic Genes 

Existing gene therapies for mtDNA mutations are currently confined to just one organ (the eye). The common problems of the gene therapy field apply; namely, (1) safe integration, (2) prolonged expression, and (3) wide tissue distribution. AE might have an additional problem: in some cases, more than one gene needs to be transferred. Furthermore, mtDNA mutations are pleiotropic and can affect almost all tissues in the body, although particular tissues tend to be most affected in mitochondrial disease. One strategy to overcome these limitations is by introducing the foreign genes at sheltered locations in the nucleus via TALENS or CRISPR/Cas9. Although such studies have not yet reached clinical phases, advances in identifying safe harbor loci in the human genome have facilitated in vitro transgene expression for large genes, such as the human dystrophin [169] and beta-hexosaminidases in Tay-Sachs and Sandhoff’s syndromes [170], as well as the expression of multiple glycosyl hydrolases in the CEP112 locus in animal models [171]. Another alternative is to place such transgenes in human artificial chromosomes (HACs) capable of large insertions that can be maintained episomally under physiological conditions [172].

The gene therapy field almost exclusively uses AAV vectors, serotypes 2 and 5, because of their high infectivity in a broad range of cell types and tissues. However, therapeutic use of AAV is limited by insert capacity (<5 kb) [173], which prevents inclusion of more than one gene in these viral vehicles. This is more evident when the mutation affects mtDNA transcription or translation, such as mutations in the origin of replication for the light and heavy strands [174], or in any of the tRNA genes that impact global protein translation in the mitochondrial matrix [175]. Under such circumstances, achieving meaningful therapy may therefore require transgene expression for more than one gene or, in certain instances, for those of all 13 mtDNA protein subunits. Alternative delivery systems using adeno- or lentiviruses or using multiple viruses with different cargos could be considered. Beyond the current scope of prospective gene therapies, strategies such as mini-chromosomes or safe harbor integration directly into the nuclear genome have the potential capability of introducing >100 kb at a time, though there is not yet, to our knowledge, precedent for delivering such large cargos into clinically viable vectors.

## 8. Allotopic Expression Gene Therapy in Human Clinical Trials

Translational advances have also been made in isolated physiological compartments, such as the case of intravitreal injections for treatment of LHON in human patients by the gene therapy company GenSight Biologics SA, and separately in two small unaffiliated academic studies [176] (ClinicalTrials.gov, Identifier NCT02161380), all of which reported anecdotal improvements in vision in treated LHON patients harboring mtND4 mutations. Specifically, these studies were able to follow patients for 9–36 months after intravitreal injection of an rAAV2–ND4 gene therapy vector and fluctuations in visual acuity were assessed. The therapy has been shown to be safe and improvements in symptoms were observed in many patients. GenSight Biologics has also completed two phase 3 clinical trials to treat blindness caused by mtND4 mutations (ClinicalTrials.gov, Identifiers NCT03293524 and NCT02064569) and recently reported sustained positive results from the RESTORE study [177], though at the time of this writing neither study has reported results in any clinical trial registries. The studies aimed for internal controls where one eye was treated and the other not, but surprisingly patients appear to have recovered vision in both eyes. There is reason to believe that the viral vector and/or the mitochondria migrated from one eye to the other to yield the benefit, but as the studies lack any meaningful placebo control, it is difficult to conclude efficacy with high confidence. GenSight Biologics has now initiated a third phase 3 trial in which one arm of the study will receive the same single eye treatment while the other arm receives only a mock treatment. The trial is expected to conclude in 2024 (ClinicalTrials.gov, Identifier NCT03293524). At the time of this writing, Wuhan Neurophth Biotechnology is also recruiting for a phase 1/2/3 clinical trial of an ND4 gene therapy for LHON patients expected to conclude in 2027. For LHON patients suffering from vision loss, the promising results of these clinical trials could be a first-in-class targeted gene therapy for a single gene mtDNA disease. Furthermore, as both gene therapies and allotopic expression technologies advance, such precedent could lead to broader therapeutic applications for both genetic diseases and aging.

## 9. Conclusions

Mitochondria are at the interface between several critical functions in the cell, including metabolism, signaling, and immune surveillance. Advances in our understanding of mitochondrial biology and function have illuminated the role of mitochondrial dysfunction in pathology and aging. The unique properties of the organelle predispose its genome to mutations and compromised functions leading to several diseases collectively called mitochondriopathies. Researchers have exploited various technologies, including small-molecule drugs, allogeneic hematopoietic stem cell transplantation, mitochondrial replacement, as well as gene-editing tools, such as nucleic acid therapy and mitochondria-targeted restriction endonucleases, in alleviating these diseases. While modulating organelle function using small molecules is attractive at the outset and benefits from ease of administration, few leads have been identified that hold curative promise, and this treatment modality leaves the root cause of pathology unaddressed. Compounds currently in clinical trials are predominantly antioxidants (such as Idebenone, EPI-743 and RPI-103) and small molecules that have the capability of stabilizing the organelle membrane architecture (Elampritide/SS31) [178]. Cell and, particularly, organelle replacement approaches are being explored in the fertility space in circumventing transmission of pathogenic variants to progeny, but the efficacy and ethical considerations pose major concerns.

Recent gene editing approaches, such as targeted restriction endonucleases and base-editing enzymes show promise, though they are limited by their narrow specificity and may require patient-to-patient customization. Gene therapy in the form of allotopic expression has received the most attention for its potential as a robust method for reversing the symptoms of mtDNA mutations. Synchronizing allotopic expression for the 13 mtDNA genes with the nuclear-mitochondrial transcription and translation machinery can overcome limitations in competing with pre-existing mutant proteins in the respiratory chain complexes due to heteroplasmy, a condition commonly observed in known mtDNA pathologies. Furthermore, advances in technologies capable of inserting large DNA cargos into the nuclear genome, such as safe harbor expression or mini chromosomes, will allow for testing multiple allotopic genes simultaneously. While validating the technology in vivo has its challenges due to inadequate animal models for all the protein coding genes, the ease of generating precise human iPSCs, particularly from patients with specific mtDNA mutations, may allow us to test these gene therapy approaches on a case-by-case basis in vitro. At the organ level, the immune privilege of the eye allows for many gene therapy trials to be conducted in patients suffering from mitochondriopathies of the eye, simultaneously establishing precedent for this model in evaluating translational approaches. The findings reviewed here suggest that innovative molecular and genetic therapies targeting mtDNA may soon become available.

## Figures and Tables

**Table 1:** Allotopic expression studies and experimental strategies.

Expressed mtDNA Gene	Expression System	Gene Origin	Strategy Features			References
			MTS	Gene	Other	
ATP6	*S. cerevisiae*	*S. cerevisiae*	+ *	Optimized	---	[106,107]
ATP6	*S. cerevisiae*	*S. cerevisiae*	++ *	Optimized	---	[108]
ATP6	*S. cerevisiae*	*P. anserine*	+, + *	Recoded	---	[109]
ATP6	Oli-sensitive CHO line (11-11); NARP cybrids (T8993G)	*C. griseus*	+	Recoded	---	[110]
ATP6	HEK293, COS7 cell lines; NARP and MILS cybrids (JCP213, JCP261)	*C. reinhardtii*	---	Unchanged	---	[111]
ATP6	HEK293, 143B WT cell lines; NARP cybrids JCP261 (206.8993E (T8993G))	*H. sapiens, C. reinhardtii*	+ *	Recoded	---	[112]
ATP6	NARP cybrids JCP261 (T8993G)	*H. sapiens*	+	Recoded	---	[102]
ATP6	HeLa	*H. sapiens*	+	Recoded	3′UTR	[113]
ATP6	NARP cybrids (T8993G)	*H. sapiens*	+	Recoded	3′UTR	[114]
ATP6	CHO; NARP cybrids (T8933G)	*H. sapiens*	+ *	Recoded	Multiple residue substitutions to reduce TM hydrophobicity	[115]
ATP6, ATP8	A8/A6 mutant cybrids (G8529A)	*H. sapiens*	+	Recoded or optimized	coexpression of ATP6 and ATP8	[116]
ATP6	Transgenic ATP6 WT or NARP/MILS mutant (L156R in ATP6) mice	*M. musculus*	+	Recoded or mutant recoded ATP6	---	[117]
ATP8	*S. cerevisiae*	*S. cerevisiae*	+, + *	Optimized	---	[98,107,118,119]
ATP8	*S. cerevisiae*	*S. cerevisiae*	+ *	Optimized	---	[108]
ATP8	HeLa, COS-7	*H. sapiens*	+, ++, + *+	Recoded	---	[99]
CYB	*S. cerevisiae*	*S. cerevisiae*	+ *	Recoded	Piecewise import as TM bundles	[120]
CYB	HeLa, COS-7	*H. sapiens*	+, ++, + *+	Recoded	---	[99]
ND1	LHON ND1 cybrids (G3460A)	*H. sapiens*	+		3′UTR	[121]
ND1	Heteroplasmic ND1 KO cybrid line (mt3571insC)	*H. sapiens*	+	Recoded	5′UTR and 3′UTR	[122,123]
ND1	HEK293 and 143B WT lines; homoplasmic ND1 KO cybrid line (mt3571insC)	*H. sapiens*	+	Recoded, optimized	---	[124]
ND4	LHON cybrids (G11778A)	*H. sapiens*	+	Recoded	---	[105]
ND4	*M. musculus* (DBA/1J)	*H. sapiens*	+	Recoded or mutant recoded ND4 (R340H)	In vivo	[125]
ND4	*M. Musculus* (DBA/1J)	*H. sapiens*	+	Recoded	In vivo	[126]
ND4	HeLa, COS-7	*H. sapiens*	+, ++, + *+	Recoded	---	[99]
ND4	LHON ND4 cybrids (G11778A)	*H. sapiens*	+	Recoded	3′UTR	[114,121]
ND4	In vivo in rat retina induced LHON model (G11778A)	*H. sapiens*	+	Recoded, optimized, or mutant recoded ND4 (G11778A)	3′UTR; IRES, β globin intron introduced into gene construct	[127,128]
ND6	Mouse NIH3T3 ND6 KO mutant line (del13887)	*M. musculus*	+	Recoded	---	[100]
COX1	HeLa, HEK293T, MCF-7, MDA-MB231	*H. sapiens*	+	Optimized	---	[129]
COX1	HeLa	*Bos taurus*	+	Recoded or mutant recoded COX1 (D51N)	---	[130,131]
COX2	*S. cerevisiae*	*S. cerevisiae*	+	Recoded	Single, double, or triple residue substitutions to reduce TM hydrophobicity	[132]
COX2	*S. cerevisiae*	*S. cerevisiae*	+	Recoded	W56R mutation to reduce TM hydrophobicity	[133,134]
COX2	*S. cerevisiae*	*S. cerevisiae*	+, + *	Recoded	3′UTR; W56R mutation to reduce TM hydrophobicity	[97]
COX3	CHO; COX3 15bp deletion cell line CSP112.5	*H. sapiens*	+ *	Recoded	multiple residue substitutions to reduce TM hydrophobicity	[115]
ATP6, ATP8, ND1, ND2, ND3, ND4, ND4L, ND5, ND6, COX1, COX2, COX3, CYB	HEK293 and 143B WT cell lines	*H. sapiens*	+	Recoded and optimized	---	[124]
mtATP6 (mRNA)	*Drosophila* model for mitochondrial encephalomyopathies (MEs)	*D. melanogaster*	---	*Drosophila* mtATP6 mRNA	mRNA targeted to mitochondrial matrix for expression (*mtTRES^Pro^*)	[135]
mtND1, mtND3, mtND4, mtND6, mtCOX2, mtCOX3, mtATP6, mtATP8 (mRNAs)	HeLa	*H. sapiens*	MTS Panel	Human mRNAs	3′UTR Panel	[104]

Strategies employed across allotopic expression studies of mitochondrial genes. Subunits listed are proteins expressed using nuclear translation machinery. Subunits preceded by “mt” (e.g., mtND4) are genes or mRNA encoding the subunit, for expression within mitochondria. Abbreviations: TM, transmembrane domain. Symbols: + single MTS, same species as origin of gene expressed; ++ double MTS, same species as origin of gene tested; + * single heterologous MTS; ++ * heterologous tandem MTS; + *+ chimeric double MTS. “Recoded” indicates minimal adjustments to the allotopic expression constructs for productive nuclear translation; “optimized” indicates codons optimized for nuclear translation; and “unchanged” indicates expressing a transkingdom gene without any modifications.

## Abbreviations

AE	Allotopic expression
AHSCT	Allogeneic hematopoietic stem cell transplantation
ALS	Amyotrophic lateral sclerosis
ATP	Adenosine triphosphate
ETC	Electron transport chain
HR	Homologous region
HSP	Hereditary spastic paraplegias
IMS	Intermembrane space
iPSCs	Induced pluripotent stem cells
AAV/AAV2	Adeno-associated virus (vector)
NADH	Nicotinamide adenine dinucleotide dehydrogenase
LHON	Leber’s hereditary optic neuropathy
NARP	Neuropathy, ataxia, and retinitis pigmentosa
MELAS	Mitochondrial encephalomyopathy, lactic acidosis, and stroke-like episodes
MERRF	Myoclonic epilepsy with ragged-red fibers
MIDD	Maternally inherited diabetes and deafness
MILS	Maternally inherited Leigh syndrome
MIM/IM	Mitochondrial inner membrane
MNGIE	Mitochondrial neurogastrointestinal encephalomyopathy
mtDNA	Mitochondrial DNA
MTS	Mitochondrial targeting sequence
OXPHOS	Oxidative phosphorylation
RC	Respiratory complex
RNS	Reactive nitrogen species
ROS	Reactive oxygen species
SCA	Spinocerebellar ataxias
TALENS	Transcription activator-like effector nucleases
TM	Transmembrane domain
UTR	Untranslated regions
